# Vegan diet: nutritional components, implementation, and effects on adults’ health

**DOI:** 10.3389/fnut.2023.1294497

**Published:** 2023-11-09

**Authors:** Edyta Łuszczki, Faustina Boakye, Magdalena Zielińska, Katarzyna Dereń, Anna Bartosiewicz, Łukasz Oleksy, Artur Stolarczyk

**Affiliations:** ^1^Institute of Health Sciences, Medical College of Rzeszów University, Rzeszów, Poland; ^2^Faculty of Health Sciences, Department of Physiotherapy, Jagiellonian University Medical College, Kraków, Poland; ^3^Orthopedic and Rehabilitation Department, Medical University of Warsaw, Warsaw, Poland

**Keywords:** bioactive compounds, health, plant-based sources, prevention, vegan diet

## Abstract

Vegan diet has emerged as a popular dietary choice for people worldwide in recent times, due to concerns such as health issues, animal rights and welfare, and the sustainability of the environment. The purpose of this literature review was to explain how a vegan diet may affect the health of adults and to point out beneficial components found in it as well as any difficulties associated with its implementation. Evidence supports that a vegan diet can reduce the risk of chronic diseases, such as type 2 diabetes, hypertension, and certain types of cancer. A well-planned vegan diet must include adequate calories and nutrients, as well as the necessary supplements, such as vitamin B12, vitamin D and EPA/DHA. Given the current growing interest in plant-based diets among the general population, it is crucial to understand both the barriers, risks, and benefits of the vegan diet among physicians, policy makers, and the general population.

## 1. Introduction

### 1.1. Topic overview

Vegan diet has become a popular diet choice for people around the world, in recent times, due to concerns such as health issues, animal rights and welfare, and sustainability of the environment (1). A recent nutrition report from Germany stated that the number of vegetarians and vegan shaved doubled from pre-COVID (5%) to post-COVID (10%) (2). The prevalence of vegans in Europe has been estimated to be between 1 and 10% (3). Over the years, research has explored the nutritional benefits of a vegan diet and its potential effects on health and well-being. A well-planned vegan diet contains only plant-derived foods, such as fruits, vegetables, legumes, grains, seeds, and nuts (4). While these natural sources offer a myriad of essential nutrients, special attention must be paid to certain key components that may be more difficult to obtain solely from plant-based sources. To prevent deficiencies and ensure overall health and well-being, particular attention must be paid to nutrients such as protein, iron, calcium, vitamin B12, vitamin D, and omega-3 fatty acids with a very long chain in a vegan diet (5). As adults cultivate a vegan lifestyle, their dietary choices can substantially affect their health (6). Many studies have identified that a well-planned vegan diet can provide numerous health benefits. Evidence supports that a vegan diet may reduce the risk of chronic diseases, such as type 2 diabetes, hypertension, and certain types of cancer (5). Research conducted in Adventists has presented convincing evidence that adequately balanced vegan diets are nutritionally sufficient (7). On the contrary, inadequate intake of critical nutrients, such as vitamin B12 and iron, can lead to anemia and other health complications if not addressed or attended appropriately (8). The plant-based diet index has been developed to assess intakes of both plant and animal foods, considering the quality of plant foods: overall plant-based diet index (PDI), healthful plant-based diet index (hPDI), and unhealthful plant-based diet index (uPDI). All indices negatively weigh animal foods, but differently weigh plant foods depending on their nutritional quality (9). Previous studies have reported that hPDI was associated with a lower risk of cardiovascular disease or type 2 diabetes (10, 11).

Central to the interest in the vegan diet lies its foundation in plant-based foods, which are abundant in a diverse array of bioactive components. These constituents contribute to the potential influence of the diet on health, especially in adults (12). Bioactive components are inherent compounds within plant foods, which extend their impact beyond basic nutrition (13). Functioning as key regulators, these bioactive components modulate physiological processes and are associated with a spectrum of positive health advantages, such as antioxidant, anti-inflammatory, and anticarcinogenic properties (13). Flavonoids have positive effects on cardiovascular health and anti-cancer properties. Carotenoids exert protective effects against several types of cancers, in addition to their benefits for vision and skin. Glucosinolates have protective roles against cancer and dementia (13).

Research has indicated that a well-planned vegan diet can confer improved health benefits while aligning with principles that favor ethical and environmental concerns (6). However, certain knowledge gaps must be explored. By extensively exploring the nutritional components of a vegan diet and their implications for well-being, individuals, especially adults, can make informed choices about adhering to a vegan lifestyle. This article analyses the essential aspects of a well-planned vegan diet, while concentrating on its nutritional components, health effects in adults, and specific nutrients that require careful consideration to ensure the maintenance of optimal health. Therefore, the purpose of this review of the literature was to explain how a vegan diet may affect the health of adults and to point out the beneficial components found in it, as well as any difficulties associated with its implementation.

## 2. Materials and methods

The article is of a narrative review nature. The main objective of this study was to review scientific publications in order to establish relationships arising from the adoption of a vegan diet among adults. Attention was focused on the beneficial components present in the vegan diet and their impact on health, the appearance of vitamin deficiencies, and the challenges associated with adhering to this type of diet.

The following source selection criteria were applied: studies related to the adoption of a vegan diet among individuals aged 40–85 years, encompassing randomized clinical trials, observational studies, meta-analyses, systematic reviews, as well as documents containing recommendations and guidelines.

General reviews, case studies, articles not published in the English language, and studies involving pregnant and breastfeeding women and athletes were excluded from the review.

The following databases were utilized: PubMed, Science Direct, Web of Science, Scopus, Google Scholar, CINAHL, Web of Knowledge, Medline, PsycINFO. The search process employed the following words, phrases, and sentences in accordance with the content and purpose of the study: “vegan diet,” “adults,” “bioactive compounds,” “health,” “plant sources,” “carotenoids,” “antioxidants.”

In total, 155 literature sources were collected.

## 3. An explanation of the vegan diet

It should be noted that the various types of diet that exist are identified and distinguished by the items that are included and excluded (12). According to Jakše et al., a vegan diet can be described as one excludes any foods derived from animals but is rich in foods from various plant-based dietary groups, such as fruits and vegetables, grains, legumes, nuts, etc. and the vegan diet is the most stringent version of a plant-based diet (4). Insights collected from previous research indicate that a carefully structured vegan diet can lead to notable health benefits, as the components of food typically contain essential components vital for well-being (4).

## 4. Substances/compounds present in vegan diet

### 4.1. Dietary fiber

Dietary fiber is an integral component of the vegan diet, offering multifaceted contributions to human health. Dietary fiber resists digestion within the gastrointestinal tract (12), providing a range of physiological benefits that encompasses the modulation of digestion, absorption of nutrients, and impact on gut microbiota (14). Depending on how well fibers dissolve in water, they can be classified as soluble or insoluble (15). Soluble fiber, found in foods like fruits, oats, and beans, dissolves in water to create viscous substances. This property contributes to slowing digestion and increasing satiety (16). On the other hand, insoluble fiber, prevalent in whole grains and vegetables, adds bulk to fecal matter, thereby facilitating regular bowel movements and preventing constipation (14). The mechanisms through which dietary fiber exerts its health effects are well-established. Soluble fiber forms a gel-like matrix within the digestive tract, delaying stomach emptying and retarding glucose absorption, thus supporting blood glucose regulation (17). Furthermore, dietary fiber has a positive influence on gut microbiota. The interaction between dietary fiber and gut microbiota involves a fermentation process. Bacterial fermentation of fibers, including resistant starch, some simple sugars, and polysaccharides, leads to the production of short-chain fatty acids (SCFAs) like acetate, propionate, and butyrate. These SCFAs play an essential regulatory role in lipid metabolism, cholesterol and glucose regulation, anti-inflammatory responses, immune functions, and the maintenance of the integrity of the gut barrier (18, 19). These SCFAs create an environment conducive to the growth of beneficial bacteria such as *Lactobacillus* and *Bifidobacteria*. These bacteria thrive on SCFAs and utilize saccharide metabolism to compete effectively with harmful bacteria for resources (18). These microbial byproducts, along with other bioactive compounds formed through the fermentation or degradation processes driven by gut microbiota, not only provide vital sustenance for the growth of gut microbes but also exhibit the capacity to influence diverse pathways within the intestines, liver, and pancreas, thereby contributing to the overall enhancement of gut health (20).

SCFAs offer a broad spectrum of health-enhancing effects, acting as anti-inflammatory agents by regulating cytokine production and immune cell functions. In colon cancer cells, they demonstrate properties that counteract carcinogenesis by influencing acetylation and activating G protein-coupled receptors (GPCRs); they also share associations with microRNAs and Vascular Endothelial Growth Factor (VEGF) (19). In particular, SCFAs play a vital role in preserving the integrity of the intestinal barrier by coordinating the expression of tight junction proteins. Furthermore, they contribute to alleviating oxidative stress by regulating oxidoreductase activity, promoting nuclear factor erythroid 2-related factor 2 (Nrf2), and reducing reactive oxygen species (ROS) and reactive nitrogen species (RNS) levels (19). Disruption in the equilibrium of the gut microbiota can result in impairment of the intestinal barrier, thereby increasing susceptibility to particular diseases (21). Simultaneously, microbial byproducts like short-chain fatty acids and other bioactive constituents, generated through the fermentation or breakdown processes facilitated by gut microbiota, serve as essential nourishment for the proliferation of gut microbes. Furthermore, these compounds have the potential to influence various pathways within the intestines, liver, and pancreas, ultimately contributing to improved intestinal health (22).

Based on findings from a study exploring the association of dietary type with fecal microbiota and short chain fatty acids in vegans and omnivores, it was observed that individuals following the vegan diet showed a distinctive composition of gut microbiota, characterised by increased amounts of *Firmicutes* and *Bacteroidetes* (23). In the study by Reiss et al., *Firmicutes* and *Bacteroidetes* were shown to make up about 95–97.7% of the total gut microbiota in vegans and omnivores, with *Firmicutes* contributing around 59% and *Bacteroidetes* contributing 39% to the total gut microbiota in vegan (23). Compared to omnivorous diets, vegan diets are generally richer in fiber and contain less saturated fat and protein. Long-term vegetable intake has been shown to correlate with gut microbiota diversity, and higher fiber intake increases the prevalence of microorganisms associated with a healthy gut (24).

Another study investigated the interaction between dietary fiber intake and microbial diversity within diverse diet patterns, including the vegan diet. Although there was no notable variation in microbial abundance between diets, the study found that people following a vegan diet exhibited reduced microbial diversity compared to those consuming a western diet. Subsequent analysis of beta diversity indicated discernible differences in the composition of gut microbiota, regardless of fiber intake levels. Notably, within the vegan group, both high and low fiber intake led to unique microbial compositions compared to the Western Diet. These findings underscore the collaborative impact of diet composition and fiber intake on the diversity of the gut microbiota, highlighting the need to consider both aspects in understanding interactions between diet and gut microbiota (25). The study emphasizes the importance of considering diet quality, aligning with a research that underscores the role of incorporating whole foods and dietary fiber to foster improved and holistic health results (8).

Consequently, this mechanism forms a protective barrier against the invasion and proliferation of pathogenic bacteria, contributing to the establishment of a healthier and more balanced gut microbial community (5). Insoluble fiber, due to its bulk-enhancing effect, accelerates intestinal transit and prevents constipation, functioning through the mechanical stimulation of regular bowel movements and the maintenance of gut motility (26). The role of dietary fiber in mitigating constipation has been established. A randomized controlled trial that administered a “vege powder” comprising whole grains, broccoli and chicory to constipated participants showed improved symptoms, including increased defecation frequency, softer stool, and reduced strain (14). Additionally, dietary fiber has demonstrated efficacy in enhancing insulin sensitivity and overall metabolic health. According to Barber et al., an observational study highlighted an association between increased dietary fiber ratio and a significant reduction in HbA1C, when compared to general carbohydrate intake (14). A double-blind, randomized, placebo-controlled trial suggested the potential for fiber as a complement to lifestyle and/or pharmaceutical interventions for managing type 2 diabetes including improved HbA1c (27). Dietary fiber offers an array of critical functions within the context of a vegan diet. Its contributions include weight management, lowered cholesterol levels, stabilized blood glucose levels, and improved digestive health (4). A randomized controlled trial comparing the effectiveness of different plant-based diets for weight loss investigated how various diets influenced weight loss and dietary behaviors, revealing significant insights. Diets did not emphasize caloric restriction. During 6 months, participants adhering to a vegan diet exhibited the most substantial weight loss (−7.5% ± 4.5%), surpassing the other diet groups. This weight loss trend was statistically significant at 2 months (*p* < 0.01) and 6 months (p < 0.01). Notably, the vegan group showed significant increases in fiber intake compared to other groups at 2 months (p < 0.01), underscoring the potential impact of dietary fiber, particularly within vegan diets, on weight loss outcomes. These results underscore the importance of incorporating fiber-rich diet choices, such as those of a vegan diet, to promote successful weight management (28).

Furthermore, in a prospective study investigating the effects of changes in the diet pattern on diabetes risk, the exclusion of individuals with existing diabetes, elevated fasting blood glucose, a history of cancer, coronary heart disease, stroke, and alcohol / cigarettes led to a final analysis of 2,918 participants. Within this group, nonvegetarians demonstrated higher BMI, waist circumference, and fasting blood glucose levels. During a 5.2-year period, 183 participants developed diabetes. In particular, both people who consistently followed high-fiber diets and those who recently switched to such diets exhibited a remarkable 40–60% decrease in risk of diabetes compared to non-vegetarians. This protective effect of embracing a high fiber diet was particularly pronounced among those with healthier metabolic profiles. The robustness of these findings was consistently supported by sensitivity analyses. Taken together, the evidence from this study underscores the potential of adopting a high-fiber diet, whether through ongoing adherence or conversion, to significantly mitigate the risk of diabetes (29).

This makes a vegan diet particularly advantageous for adults who may be susceptible to various chronic diseases and disorders.

### 4.2. Bioactive components

Plants contain essential minerals necessary for a well-balanced human diet, along with other primary and secondary metabolites that affect both nutrition and health (12). Plant foods serve as abundant reservoirs of nutrients such as magnesium, calcium, potassium, minerals, and vitamins, and bioactive elements such as polyphenols, dietary fiber, and carotenoids. For antioxidants, their health benefits have been well established. Antioxidants are compounds in plants that counteract the harmful effects of oxidative stress, protecting cells from free radical damage. They can be obtained from the phenolic components of plants (12). The role of antioxidants in mitigating damage caused by free radicals has been extensively studied. The consumption of a large number of plant-based foods in the diet reveals this significant benefit (30). Consuming plant foods rich in antioxidants has been associated with reduced risk against various diseases such as cancer, cardiovascular disease, and type 2 diabetes (30). A study conducted by Miller et al. (2017) investigated a cohort of 135,335 younger and older adults from 18 different countries. The findings of this study indicated a significant link between a diet rich in fruits, vegetables, and legumes and a decreased susceptibility to cardiovascular-related mortality. The observation of this study implies that the phytochemicals in these plant-based foods could play a role in mitigating diabetes and decreasing susceptibility to cardiovascular diseases. These potential mechanisms include the regulation of α-glucosidase and lipase activities, the postprandial decrease in blood glucose levels, anti-inflammatory effects, enhancement of pancreatic function, and potential synergistic interactions with hypoglycemic medications (30). Vegan diets play a vital role in counteracting the detrimental effects of free radicals and ROS by harnessing robust antioxidant capacities. Abundant in antioxidants, the vegan diet effectively combats harm related to oxidative stress, promotes cellular health, and reduces susceptibility to chronic diseases (31). Antioxidants also regulate immune processes, reduce DNA damage, and reduce lipid peroxidation, all of which contribute to better health (13). By donating electrons, antioxidants disrupt the chain reaction of oxidation, effectively mitigating cellular damage (26). Within a vegan diet, a variety of diverse antioxidants, including vitamins C and E, polyphenols, and carotenoids, collaborate synergistically to counteract the formation of ROS arising from multiple origins such as metabolic processes, lifestyle activities, and environmental elements. This holistic defensive mechanism offers robust protection against the aforementioned diseases (26). In addition to scavenging free radicals, research has shown that antioxidants in fruits and vegetables are responsible for the expression of genes through transcription factors, cell signaling modulation, and enzyme activities. These antioxidant properties can be linked a reduction in oxidative stress, improved cell function, and a lower risk of chronic diseases (30). Carotenoids represent another group of bioactive compounds found in a vegan diet. Fruits and vegetables that are yellow or orange possess this bioactive compound (32).

Carotenoids are known for their diverse health-promoting characteristics, including anti-cancer, anti-inflammatory, anti-aging and anti-microbial traits (12). They eliminate free radicals by neutralising them and blocking lipid peroxidation, which is associated with cell damage (33). In particular, carotenoids serve as precursors to vitamin A, which is a crucial fat-soluble vitamin with various functions in maintaining health and immune function (34). Vitamin A is known to regulate the differentiation and proliferation of immune cells, thus affecting the immune response (33). Furthermore, it has been postulated that they can interact with nuclear receptors, such as retinoic acid receptors, influencing gene expression involved in inflammation and immune function. Through conversion to vitamin A, they are recognized for their pivotal role in the health and functionality of photoreceptor cells in the eyes, for good vision (33). In a study with 74 participants having type 2 diabetes, an 8-week fruit and vegetable (F&V) intervention significantly increased carotenoid levels, including *α*-carotene, *β*-cryptoxanthin, lutein, and zeaxanthin (*p* < 0.05). These increases, ranging from ~8% to ~25%, were evident in both serum and HDL fractions. The results suggest that elevated F&V intake may enhance HDL’s antioxidant properties through an increase in carotenoid content (35). Polyphenols are another set of bioactive compounds present in plant foods. They are distinguished by the presence of various phenol groups in their chemical structures. These naturally occurring bioactive compounds are synthesized by plants as secondary metabolites. Polyphenols can be classified into four main classes, namely flavonoids, phenolic acids, lignans, and stilbenes, according to their structural characteristics (16). Numerous *in vitro* studies have highlighted the substantial antioxidant capacity of polyphenols, a result of their facilitation of ROS neutralization. This pronounced antioxidant potential, combined with its ability to support nitric oxide (NO) production, contributes to the protection of endothelial function of polyphenols. Furthermore, polyphenols contribute to improving cardiovascular health by inhibiting platelet aggregation, mitigating vascular inflammation, facilitating apoptosis, reducing LDL cholesterol oxidation, and improving lipid profiles (16, 36). Investigating the effects of bioactive compounds, a study explored the potential cardiovascular benefits of olive oil polyphenols, yielding significant insights into LDL-related health outcomes. This 3-weeks randomized controlled trial in Men investigated the effects of olive oil polyphenols on health. The intervention led to reduced LDL concentrations, including apolipoprotein B-100 (apo B-100) levels a crucial component of LDL, which has a history of being associated with atherosclerosis and cardiovascular risk, making the reduction in apo B-100 levels a significant finding and total LDL particles, compared to low-polyphenol olive oil (*p* = 0.004 and *p* = 0.013, respectively). Notably, the high-polyphenol olive oil intervention also lowered the number of small LDL particles (*p* = 0.029) and increased LDL resistance against oxidation (*p* = 0.038), suggesting potential cardiovascular benefits by improving LDL-related health outcomes (37).

Another study was conducted to compare polyphenol intakes according to different food patterns and food sources in the Adventist Health Study-2 cohort. Among the dietary groups examined, vegans emerged as having the most substantial polyphenol intake. This elevated intake among vegans was statistically significant (*p* < 0.05). These observations emphasize the potential of a vegan dietary approach to offer increased polyphenol consumption, underscoring its relevance in the intake of bioactive compounds (38). Furthermore, exploring the effects of polyphenols, a study investigated the influence of strawberry and cranberry polyphenols (SCP) on parameters such as insulin sensitivity, glucose tolerance, insulin secretion, lipid profile, inflammation, and markers of oxidative stress in individuals with insulin resistance, overweight, or obesity. The trial included participants with similar baseline characteristics. In particular, the SCP group demonstrated a significant 14% increase in insulin sensitivity (*p* = 0.05), while the control group showed a nonsignificant decrease of 7% (*p* = 0.28). This improvement in insulin sensitivity within the SCP group was statistically significant in contrast to the control group (*p* = 0.03). Although the responses to glucose, insulin, and free fatty acids aligned between the groups, the SCP beverages contributed to higher levels of specific phenolic metabolites in plasma. Of particular interest was the discovery of a significant negative correlation between plasma p-coumaric acid concentration and changes in the response to C-peptides during the oral glucose tolerance test (OGTT) (*p* = 0.0046, r^2 = 0.34). This observation suggests a potential association between different phenolic metabolites arising from SCP consumption and the improvement of insulin sensitivity (39). Another study also highlighted the effects of polyphenols on health. The objective was to investigate the effects of polyphenols and omega-3 fatty acids (LCn3) on various health markers. To achieve this, participants were randomly assigned to one of four nutritional isoenergetic interventions for 8 weeks: a control diet low in LCn3s and polyphenols; a diet rich in LCn3s and low in polyphenols; a diet rich in polyphenols and low in LCn3s; or a diet rich in both LCn3s and polyphenols. Throughout the controlled diet interventions, the experimental groups showed significant variations in polyphenol and LCn3 content. The baseline characteristics were consistent among all groups. Fasting levels of lipids, cholesterol, and glucose did not show notable differences. Body weight remained steady throughout the intervention, with slight reductions in the high-LCn3 group. Waist circumference changes were insignificant. Polyphenols appeared to significantly lower fasting triglycerides and cholesterol in larger lipoprotein fractions, while omega-3 fatty acids (LCn3) did not produce significant effects. After meals, lipid and lipoprotein levels improved, mainly due to the impact of polyphenols on triglycerides. Furthermore, markers of oxidative stress, such as 8-isoprostane, decreased significantly in the high-polyphenol groups. Interestingly, the effects of diets naturally rich in polyphenols and/or marine LCn3s on urinary 8-isoprostane were correlated with changes in plasma lipoproteins, particularly in polyphenol-rich groups (39, 40).

Polyphenols have the ability to modulate enzyme and anticancer activities and metal chelation, all of which contribute to their intrinsic characteristic of reducing the occurrence of chronic diseases (41). They prevent cytokines from binding to cancer cells and prevent oxidative stress, thus protecting against cancer (42). Certain types of polyphenols found in varieties such as green and black tea exhibit the ability to inhibit the growth of harmful bacteria such as *Helicobacter pylori, Listeria monocytogenes, Staphylococcus aureus, Escherichia coli, Salmonella typhimurium,* and *Pseudomonas aeruginosa* (20).

Another substance, ferulic acid, is a phenolic acid of the polyphenol family, found more commonly in plant-based foods such as cereals, fruits, and vegetables. It has antioxidant qualities that can help prevent many chronic diseases (36). Anthocyanins have also been found to have great antioxidant properties. They support health by controlling metabolic syndromes and neurological diseases (12). Anthocyanins exhibit the ability to form complexes with metal ions, reducing the catalytic influence of active metal ions on the generation of free radicals. This interaction contributes to an elevated antioxidant effect. In addition, anthocyanins can form complexes with copper, effectively inhibiting the oxidation of LDL induced by copper or proxy radicals (41). Vitamin C (ascorbic acid) and vitamin E (tocopherols) represent two potent antioxidants obtained primarily through the consumption of fruits and vegetables (12). Vitamin C is recognized for its involvement in cellular signaling, programmed cell death (apoptosis), and the maintenance of cell growth. Vitamin C is also a premier water-soluble antioxidant – it also functions to recycle vitamin E (e.g., reduction to active form). Vitamin E, a fat-soluble micronutrient, is involved in DNA protection and has been suggested to play a pivotal role in mitigating the reactivity of redox-active metals, such as copper and iron. In addition, vitamin E contributes to protecting against lipid peroxidation, which exemplifies its multifaceted functions in maintaining cellular health (34).

## 5. The health benefits of a vegan diet

### 5.1. Overweight and obesity prevention

Numerous studies have shown how vegan diets affect body composition, particularly when it comes to losing weight (31). Vegan diets likely lead to weight loss because they are associated with a reduced calorie intake due to a lower fat content and a higher dietary fiber content. Calorie density is very important for reducing body weight. Consuming foods with lower calories is more advantageous for weight loss than reducing portion sizes (30). Foods of plant origin have a lower calorie density than foods of animal origin (43). Data from the Adventist Health Study (AHS) have shown that the body mass index (BMI) increases as the amount of animal foods in the diet increases (44). Furthermore, results from the European Prospective Investigation into Cancer and Nutrition – Oxford (EPIC-Oxford) study have shown that vegans gain significantly less weight as they age compared to omnivores (45). The European Prospective Investigation into Cancer and Nutrition-Physical Activity, Nutrition, Alcohol, Cessation of Smoking, Eating Out of Home and Obesity (EPIC-PANACEA) study, found a positive association between total meat consumption and weight gain, even after adjusting for energy intake: an increase in 250 g/day of meat led to a weight gain of 2 kg after 5 years (95% CI, 1.5–2.7 kg) (46). In a study that examined a cohort of 49,098 Taiwanese adults, the percentage of participants with a BMI ≥ 27 kg/m2 was significantly lower among those following a vegetarian diet (10.9%) as compared to those following a non-vegetarian diet (15.4%). Furthermore, this study also found that for each year on a vegan diet, the risk of obesity decreased by 7% (47).

Vegan diets have been shown to have the lowest calorie density and the least amount of cholesterol among various plant-based diets (41). According to a study conducted by Kahleova et al. overweight participants with a body mass index between 28 and 40 kg/m2 were randomly assigned to follow a low-fat vegan diet in a randomized clinical trial (48). According to the study findings, the overweight group assigned to a vegan diet experienced a considerable decrease in fat mass and visceral fat compared to the control group. Research has shown that a vegan diet can help adults lose weight by replacing high-calorie items with low-calorie alternatives and maintaining a balance between energy intake and energy expenditure (40). Based on available evidence, the vegan diet should be considered a viable option for patients who are interested in preventing overweight and obesity or losing weight. Researchers report that a vegan diet is generally associated with a healthy lifestyle that excludes smoking and includes regular physical activity. It is very likely that the health benefits from such nutritional behavior are the result of the combination of these factors, and not only the diet alone. Such a lifestyle provides many benefits and can prevent some chronic lifestyle-associated diseases, including obesity and cardiovascular diseases (CVD).

### 5.2. Cardiovascular diseases (CVD)

Cardiovascular disease is a major cause of mortality and is currently responsible for a third of all deaths worldwide (1). CVD is a collection of different conditions that are directly related to the health of the heart. These include arteriosclerosis, arterial stenosis, arterial thrombosis, coronary heart disease and high blood pressure (49). Plant-based diets reduce CVD risk factors, as confirmed by a meta-analysis and systematic review of prospective cohort studies by Quek et al. in which they show a beneficial effect of plant-based diets in terms of reducing cardiovascular mortality and CVD (50). However, specifically in the context of a vegan diet, the systematic review by Kaiser et al. evaluated the usefulness of vegan diets in the prevention of cardiovascular disease (51). The evidence among the Western populations studied weakly suggests an association between vegan diets and the risk of cardiovascular disease. The risk of total CVD, coronary heart disease, acute myocardial infarction, primary stroke, haemorrhagic stroke and ischaemic stroke was assessed. None of the trials found a significantly increased or decreased risk of any cardiovascular complication in people who followed a vegan diet. The authors noted that due to the limited number of high-quality studies, the overall evidence on the role of a vegan diet in the development or prevention of CVD is weak (51). In this year’s systematic review and meta-analysis of prospective cohort studies, Dybvik et al. observed an 18% reduction in the relative risk of ischaemic heart disease among vegans. No clear association was observed between vegan diets and CVD or stroke. The authors indicated that the number of studies was limited and the associations unclear and imprecise (52).

Benefits of vegan diets can include reduced inflammation, blood pressure, total cholesterol, serum glucose, improved endothelial function, reduced risk of blood clots, reduction in body weight, etc. (53, 54). These beneficial cardiovascular health results can be attributed to a lower intake of dietary cholesterol, saturated fat, trans fatty acids, processed meat, and a higher and more regular intake of fibre, vegetable protein, beta-carotene, vitamin C, vitamin K, folic acid and magnesium and potassium (53). The study by Pickering et al. highlights the importance of potassium and magnesium for the health of the cardiovascular system (55). The main role of potassium in the prevention of cardiovascular disease is due to its influence on maintaining electrolyte balance, reducing blood pressure, and the risk of stroke through its beneficial effects on endothelial function and vascular homeostasis, while magnesium is involved in the regulation of blood pressure and metabolism. It is worth noting that the balance between potassium and sodium is of crucial importance for the health of the cardiovascular system (56).

In particular, the soluble fiber fraction, through its effects on lowering total cholesterol and low-density lipoprotein (LDL) levels, better glycaemic control, weight loss, and reduced inflammation, provides a number of health benefits in the context of CVD risk reduction (57). According to Pereira et al. an increase in soluble fiber intake of 10 g per day can reduce the risk of a coronary event by 14% and the risk of coronary death by 27% (58). Furthermore, the above fiber fraction may have an effect on the production of short-chain fatty acids (SCFAs) in the colon, which in turn may potentially have an effect on cholesterol synthesis (59). Nevertheless, it is worth noting that in the systematic review and meta-analysis by Wang et al. vegetarian diets were effective in lowering blood levels of total cholesterol, low-density lipoprotein cholesterol, high-density lipoprotein cholesterol, and non-high-density lipoprotein cholesterol to a greater extent than control diets; however, it is unclear whether vegan diets have a similar effect (60). A vegan diet seems to exhibit greater efficacy in reducing overall and LDL cholesterol when compared to omnivorous control diets; nevertheless, its impact on HDL cholesterol and triglyceride levels remains inconclusive (61).

Vegan diets are also rich in polyphenols, which is relevant in relation to cardiovascular disease. The antioxidant capacity of polyphenolic compounds is known from *in vitro* studies, mainly through their role in the capture and neutralization of free oxygen and nitrogen species and protection against oxidative stress. This antioxidant capacity, possibly together with their ability to modulate nitric oxide (NO) production, enables polyphenolic compounds to contribute to the maintenance of vascular homeostasis. Through their role in inhibiting platelet aggregation, reducing vascular inflammation, modulating apoptotic processes, reducing LDL oxidation, and improving the lipid profile, polyphenols can also contribute to cardiovascular health (62).

The gut microbiome is another emerging pathway through which a healthy plant-based diet may influence the risk of CVD (63). Microorganisms in the gut metabolize a variety of dietary substrates, which can have an impact on cardiovascular health (64). The trimethylamine N-oxide (TMAO) pathway is an example. Choline and L-carnitine, compounds derived mainly from animal-based foods such as red meat, poultry, and fish, are broken down by microbes in the gut to produce trimethylamine (TMA), which is further broken down in the liver to form TMAO. Associated with an increased risk of cardiovascular events, TMAO is believed to affect heart health through cholesterol and sterol metabolism, inflammation, thrombotic, and atherosclerotic pathways (65). As a recent study did not find an association between TMAO and dietary factors, it is possible that the association of animal foods with heart disease risk through the TMAO pathway is modified by eating foods rich in TMAO precursors and by gut microbial composition (66). Certain phytochemicals (e.g., resveratrol) have been found to potentially inhibit TMAO production in animal model studies (67). Plant-based diets also differ from animal-based diets in several other microbiota-dependent metabolic pathways, including increased metabolism of dietary fiber and polyphenols, and decreased metabolism of bile acids and amino acids, which may mediate links to cardiovascular disease. To elucidate the likely complex pathways by which diet interacts with the intestinal microbial environment to influence cardiovascular health, larger studies with longer follow-up and repeated assessment of diet and microbiome are needed.

There are also limitations and risks associated with following a vegan diet for cardiovascular health, especially if the diet is poorly balanced, as vegans may have lower amounts of dietary nutrients such as eicosapentaenoic acid (EPA) and docosahexaenoic acid (DHA), selenium, zinc, iodine, iron, calcium and vitamin B12, vitamin D, compared to non-vegans, which can lead to adverse cardiovascular effects (68, 69). Van Winckel et al. stress that it is important to understand that both an unhealthy diet and a vegan diet can induce chronic inflammation, if the vegan diet contains insufficient amounts of nutrients and omega-3 fatty acids (70). One of the many issues in the context of vitamin B12 deficiency in a vegan diet is the risk of leading to hyperhomocysteinemia. As a result of reduced vascular elasticity and altered homeostasis, elevated levels of homocysteine induce vascular endothelial impairment. This is an important risk factor for CVD (44). It is also worth mentioning the problem of consuming large amounts of processed plant products in a vegan diet, meat substitutes and dairy substitutes, which can be high in sugars, salt, and *trans* fatty acids (71, 72).

Most short-term studies on vegan diets do not provide accurate data on long-term effects on cardiovascular health, based mainly on changes in biomarkers. Following a vegan diet also brings about a number of health benefits in terms of cardiovascular disease, but is also associated with the risk of nutrient deficiencies. It seems that a well-balanced vegan diet, rich in high-quality plant-based foods such as whole grains, fruits, vegetables and nuts, based on unprocessed products, together with supplementation (for example, an algae-based DHA supplement in addition to regular consumption of sources of ALA and vitamin B12, vitamin D) may be considered a suitable route to the prevention of cardiovascular disease, but more research on this issue is needed (49, 62). Although it requires more research and a personalized diet approach, a vegan diet may not only benefit heart health, but may also have the potential to regulate blood glucose levels and manage diabetes mellitus.

### 5.3. Diabetes mellitus

A chronic metabolic disorder called diabetes mellitus is characterised by persistently high blood glucose levels, insulin resistance, and insufficient amounts of insulin compared to physiological requirements. Due to the decreased sensitivity of the body to insulin hormone, which controls blood glucose levels, and the pancreas’ insufficient ability to produce enough insulin to compensate for this resistance, this syndrome develops (73).

The global diabetes prevalence in 20–79 year olds in 2021 was estimated to be 10.5% (536.6 million people), rising to 12.2% (783.2 million) in 2045. Just over half a billion people are living with diabetes worldwide which means that over 10.5% of the world’s adult population now have this condition (74, 75).

Because a healthful, well-planned vegan diet may be inclusive of entirely whole plant foods such as fruits, vegetables, legumes, whole grains, nuts, and seeds, which are excellent sources of dietary fiber, it naturally contains a lot of fiber (1). Soluble dietary fiber can improve glycaemic control by delaying the process by which food leaves the stomach, resulting in slower glucose uptake and absorption (76). It is well established that a vegan diet can tackle important pathophysiological processes related to beta cell dysfunction and insulin resistance. A 16-week randomized controlled experiment with 75 overweight adults, half of whom followed a vegan diet, and the other half a control diet, illustrates this. The vegan group demonstrated a notable improvement in beta cell function and fasting insulin sensitivity compared to the control group. These two elements are recognized to be the main pathophysiological mechanisms driving type 2 diabetes (77). Another study has shown that fiber helps delay the absorption of glucose in the gastrointestinal tract, which causes blood glucose levels to gradually rise. This result may reduce the likelihood of insulin resistance and hyperglycemia (78). A study by Chester et al. showed that a low-fat vegan diet resulted in improved glycaemic control and decreased medication consumption in those with type 2 diabetes over the age of 50 years (79). According to the study, the weight loss effect of the vegan diet may account for a sizable amount of its effects on hemoglobin A1C levels, a measure of blood glucose control over time. A low-fat vegan diet was found to significantly improve glycaemic control in a 22-week randomized clinical trial (79), which included people with type 2 diabetes. In particular, the study found that the A1C readings in the vegan group dropped noticeably more than those of the other diet group. In a 12-week randomized clinical trial by Lee et al., participants diagnosed with type 2 diabetes were randomly assigned to follow a vegan diet or a conventional diet. Both diets led to lower HbA1c levels, but glycaemic control was better with the vegan diet (0.3–0.6% greater reduction) than with the conventional diet (80).

### 5.4. Cognitive function

Quercetin, which is only found in plant foods, may be responsible for the effects of a vegan diet on the reduction of anxiety and/or depressive symptoms (81). Quercetin can act as a natural antidepressant by inhibiting the activity of monoamine oxidase (MAO), an enzyme that breaks down mood-regulating neurotransmitters such as serotonin, dopamine, and norepinephrine (82), resulting in higher levels of these neurotransmitters in the brain (83). This impact might reduce the signs and symptoms of anxiety and despair.

Nutrition plays an increasingly important role in maintaining optimal brain function as people age (84). Studies have shown a protective effect of a vegan diet against Alzheimer’s (84). Alzheimer’s disease is a neurological disease that typically develops with increasing age and is known to be the leading cause of dementia worldwide (84). It is defined by a steady deterioration in cognitive abilities, including memory, reasoning, and behavior. The effects of diet and lifestyle choices, including a vegan diet, on Alzheimer’s disease have received much attention from researchers (85).

Part of the protective mechanisms of a vegan diet could be attributed to its beneficial effect on the reduction of inflammatory markers in dementia and Alzheimer’s disease. Meat-based dietary patterns appear to be positively correlated with biomarkers of low-grade inflammation, whereas vegetable- and fruit-based diets are inversely correlated (86). Studies providing data on biomarkers of inflammation in vegans, however, are few and inconsistent. Menzel et al. found no significant differences in any of the seven inflammatory biomarkers measured. Participants who followed a vegan diet for more than 4.8 years were more likely to have lower hsCRP levels compared to those who followed a vegan diet for less than 4.8 years (87). This may suggest that diet length may be an important factor in reducing systemic inflammation. Šebeková et al. also found that plasma CRP levels were not significantly different between vegans and omnivores (88). In the other hand, Franco de Moreaes et al. identified lower values of inflammatory markers, CRP and TNF-α/IL-10 ratio in strict vegetarians compared to vegetarians and omnivores (89). Lastly, a recent meta-analysis showed that vegans have lower CRP levels than omnivores (90).

With the popularity of veganism rising rapidly, there is an increased need for scientific study to determine how a vegan diet affects human health, particularly in relation to cognitive functioning. A low-risk lifestyle adjustment that can help maintain cognitive function and prevent cognitive ageing is to switch to a vegan diet (62). Further research is needed to prove that a vegan diet can help prevent or counteract inflammation and subsequently help reduce the risk of Alzheimer’s disease.

### 5.5. Bone wellness

Numerous health problems have been associated with adulthood and ageing, and the severity of these problems depends on various circumstances. A study by Rodrigues et al. found that ageing is related to a loss of bone mass, increasing the incidence of fractures with age (91). Osteoporosis is a degenerative skeletal condition that can increase the susceptibility of a person to fractures, especially in the hip, spine, and wrist (92). Key characteristics of osteoporosis include low bone mass and decreased bone mineral density. The health of an adult is greatly influenced by its diet, which is one of the key determinants. A vegan diet has some consequences, according to several studies conducted in the context of food. Bone health is one example. Bone health problems, which often develop with age, are substantially more common in women than in men (93). In general, high bone mineral density is preferred since it has a negative correlation with the risk of fragility fractures, especially in female adults. In other words, the lower the risk of fractures caused by decreased bone strength, the higher the concentration of bone mineral (94). Adopting a vegan diet can raise concerns about inadequate nutrient intake, which can eventually lead to lower bone mineral density (BMD) (95). Certain nutrients, such as vitamin B12, vitamin D, calcium, and omega-3 fatty acids, may be insufficient in a vegan diet. This is so because, as cited by Richter et al. and Menzel et al., these nutrients are commonly present in animal products (96, 97). Vegans showed lower bone mineral density than omnivores in a variety of bone locations, including the hip, femoral neck, and lumbar spine, according to a cross-sectional study conducted by Menzel et al. (97), which included 36 vegans and 36 omnivores, as well as adults. Furthermore, compared to omnivores, vegans exhibited lower levels of calcium, vitamin D, and vitamin K. However, the bone turnover markers of the two groups did not show appreciable variations. The study findings indicated that vegans should consume enough calcium, vitamin D, and vitamin K to maintain strong bones because they may be more susceptible to osteoporosis and bone fractures. The study also emphasized how crucial nutrient balance is for vegan diets because vegans who consume an unbalanced diet run the risk of depleting many nutrients. Vitamin D, which is often obtained by exposure to sunlight but can also be found in foods such as fatty fish, egg yolks, and the liver, is crucial for the health of bones (98). The relevance of nutritional practices as a modifiable factor that affects bone mineral density has been acknowledged (99).

### 5.6. Muscle integrity

Sarcopenia and frailty syndrome are debilitating conditions primarily associated with aging-related changes in body composition, characterized by low muscle mass and strength. These conditions ultimately lead to an increased risk of adverse health outcomes such as disability, hospitalization, or death (6, 100). It is estimated that sarcopenia affects 30% of individuals over 60 years old and 50% of those over 80 years old. Data analysis from 62 countries worldwide indicates a prevalence of frailty syndrome ranging from 12 to 24% of the population (101). This, in the face of an ever-increasing number of elderly people, is becoming a serious public health problem (77, 78). A well-planned diet is essential for older people. Although the evidence base for the role of dietary protein in maintaining good muscle health in older age is strong, the importance of protein sources is an ongoing subject of research (101–104). With the increasing number of people adopting flexitarian, vegetarian, and vegan diets, scientists are highlighting the need to pay attention to the dietary habits of older people to prevent sarcopenia and frailty syndrome. The results of the study conducted by Sotos-Prieto et al. indicate that a healthful plant-based diet was associated with lower risk of frailty whereas an unhealthful plant-based diet was associated with higher risk (105). Adequate consumption of high-quality dietary protein combined with regular physical activity is crucial to the prevention of the aforementioned conditions among older people (106). Furthermore, increasing portion sizes could be helpful in improving the intake of protein and essential amino acids (EAAs) to address the challenge of the lower anabolic properties of plant-based foods and proteins. Essential amino acids are essential components found in dietary protein that play a crucial role in maintaining muscle growth and strength throughout an individual’s lifetime. Attention should be paid to the intake of branched chain amino acids (BCAAs), especially leucine, isoleucine, and valine (107). Ingestion of dietary protein induces hyperaminoacidemia, promoting muscle protein synthesis and inhibiting muscle protein breakdown through various pathways (108). However, scientific opinions on the use of a vegan diet among older individuals and its impact on the development of sarcopenia are highly divided. According to studies by Hengeveld et al. and Tieland et al., the lower quality of protein present in many vegan meals can be problematic, especially considering that a significant number of adults struggle to meet the recommended increase in dietary protein intake (109, 110). According to Domić et al., older Spanish adults who consumed more animal protein than vegetable protein had a lower incidence of frailty. Additionally, researchers point out that several observational studies have shown a favorable correlation between animal-based protein and muscle mass and strength, indicating that a vegan diet might have negative effects on muscle mass and strength (6). The study conducted by Maroto-Rodriguez et al. provides intriguing results and a fresh perspective on the dietary habits of seniors. According to the researchers, diets with a high consumption of plant-derived foods and a lower consumption of animal-derived foods could potentially reduce the risk of frailty in elderly individuals. The study attributes positive health outcomes to the adoption of a plant-based diet, characterized by a significant intake of plant products and a lower intake of animal products. Unlike other vegetarian diets, a plant-based diet places emphasis on the quality of plant-based items. It associates the favorable impact of healthy plant-derived foods such as vegetables, fruits, whole grains, and nuts with improved health among individuals over 65, in contrast to unhealthy plant-derived products like refined grains, sugary beverages, and animal-based foods (111). The authors of the study suggest that the protective effect of a plant-based diet against frailty could be linked to the provision of essential nutrients. They highlight the antioxidant effects of vitamins C and E, carotenoids, and selenium derived from fruits and vegetables. These elements may protect against sarcopenia by reducing the exposure of muscle fibers to oxidative stress. However, biomarkers of selenium and zinc were lower in vegans, confirming that a sufficient supply of these trace elements is more difficult to achieve when following a plant-based diet (112). Additionally, the inclusion of legume and nuts protein might help prevent sarcopenia (111, 113). The authors also point out the potential anti-inflammatory effects of fruits, olive oil, unsaturated fatty acids, nuts, or coffee, which may help mitigate the low-grade chronic inflammation associated with frailty (111, 114). Similar results are presented by a Chinese study conducted among nearly 4,000 participants (115).

Researchers have shown that a vegan diet is linked to a reduced risk of frailty in men and older adults who lead a healthy lifestyle. More research is required to establish a vegan diet as a recommended dietary approach to prevent and minimize frailty among older adults. Furthermore, it should be considered to incorporate dietary interventions along with lifestyle changes to promote successful ageing, a factor that could also be significant for women (116).

Considering the substantial increase in the elderly population worldwide and the significance of the issue, future studies are needed to develop an optimal approach to nutrition and/or supplementation with isolated protein preparations for individuals who, for various reasons, choose to adhere to a vegan diet (117).

## 6. Nutritional factors to consider in a vegan diet

Some nutrients need to be taken special into account when following a vegan diet. According to a position document of the Academy of Nutrition and Dietetics (AND), a well-planned vegan diet is nutritious and can have health benefits for the prevention and treatment of various diseases. It also highlights the fact that a vegan diet should be carefully planned to ensure optimal nutritional intake. The article also highlights the particular nutrients in vegan diets that must be taken into account, such as protein, vitamin B12, vitamin D, calcium, iron, and omega-3 fatty acids (1, 118).

### 6.1. Protein

Numerous research efforts have aimed to evaluate the capacity of a vegan diet regimen to meet the appropriate protein requirements. A significant proportion of these studies contend that mean protein intake, which represents approximately 13–14% of daily caloric intake, aligns favorably with the guidelines established by the American diet recommendations (119). According to a study conducted by Alles et al., 27% of individuals following a vegan diet do not reach a minimum protein intake of 10%, thus initiating enquiries into the sufficiency of protein provisioning within the vegan diet (3). Conversely, despite a lower average protein intake in vegan diet, all studies reported in the article of Neufingerl et al. protein intake within the level of the adequate macronutrient distribution range (ie 10% E). None of the 64 studies reported protein intake below the acceptable macronutrient distribution range (AMDR) for any dietary pattern (53). In Bakaloudi *et al*’s review of 12,096 vegans, macronutrient intakes were largely adequate in vegans, with the exception of protein, where intakes were lower, slightly below the RNI (68). However, it should be noted that expert opinion is divided on the protein content of plant-based diets.

Therefore, it is essential for vegans to ensure that they get an adequate amount of proteins in their diet. Protein quality is influenced by the effectiveness of digestion and the presence of crucial amino acids. Another recommendation is to eat a variety of foods as amino acid limitation is not as serious (118). In particular, certain vegetable proteins, such as soy, exhibit enhanced digestibility, distinguishing them from the typical digestibility observed in many other plant-based foods. This concept is in alignment with the principles of the Digestible Indispensable Amino Acid Score (DIAAS), a widely used measure to assess protein quality (3). As Mariotti and Gardner highlighted, the distribution of amino acids in plant-based foods often displays a less optimal profile compared to animal-derived foods. However, it should be noted that even when adhering to a vegan diet characterised by limited diversity, achieving a considerable intake of total protein remains attainable. This can be accomplished by consuming significant amounts of plant protein foods, such as soybeans, tofu, legumes, nuts, seeds (120).

In the context of a vegan diet, the fulfilment of protein requirements is ensured through the complementary consumption of legumes and cereals, allowing individuals to obtain a comprehensive array of essential amino acids that are of paramount importance for human nutrition (14). This is especially noteworthy when considering that the Recommended Dietary Allowance (RDA) for protein intake is commonly set at 0.8 g/kg body weight (120). Advancements in modern food technology have played a crucial role in producing plant-based food products that mimic the attributes of animal-derived options. In particular, the use of soy and its derivatives has emerged as a significant strategy, allowing the achievement of satisfactory protein intake that could otherwise be difficult to achieve (15). Currently, vegans commonly include substantial amounts of legumes in their dietary patterns, a protein source that has gained attention as a potential preventive factor against ailments such as stomach, prostate, and colon cancer. Furthermore, the consumption of legumes demonstrates potential cardioprotective effects, evident through the reduction in serum lipids and lipoproteins circulating, including total cholesterol, low-density lipoprotein (LDL) and triglycerides (5).

### 6.2. Vitamin B12

Deficiencies in specific vitamins, particularly vitamin B12is significant concerns in the context of a vegan diet. Vitamin B12, a water-soluble nutrient found primarily in animal-derived foods, plays a vital role in hematopoiesis and nervous system function (5, 14). However, due to the absence of animal products, getting sufficient vitamin B12 is a challenge for vegans, leading to potentially severe deficiencies. These deficiencies can be the result of impaired absorption or inadequate intake of this essential nutrient, contributing to conditions such as megaloblastic anemia and degenerative disorders (5, 121). In particular, neurological symptoms of vitamin B12 deficiency include numbness and tingling of the hands and feet, decreased sensation, difficulty walking, loss of control of the bowel and bladder, memory loss, dementia, depression, general weakness, and even psychosis. Elevated rates of vitamin B12 deficiency, up to 80%, are observed in populations in Hong Kong and India, particularly among people following a vegan diet with limited inclusion of fortified foods or supplementation (5). As an exclusive animal-derived nutrient, vitamin B12 is absent in vegan diets, necessitating supplementation or fortified plant-based alternatives such as plant milk, cereals, and nutritional yeast (14, 122). Although the established daily recommended dietary allowance for adults in the United States is 2.4 microgrammes of vitamin B12, research suggests that due to variations in the absorption and utilization of vitamin B12 from plant sources, vegans might require higher doses of this vitamin (123). Therefore, regular monitoring of vitamin B12 levels and adaptive supplementation strategies become imperative to maintain optimal health (4).

### 6.3. Vitamin D

Vitamin D, classified as a fat-soluble micronutrient, plays a central role in promoting calcium absorption and maintaining optimal bone health (124). Its synthesis takes place on the human skin when exposed to sunlight. However, various factors, such as geographic location and time of year, can influence the skin’s ability to produce sufficient vitamin D (4, 5). According to research conducted by Melina et al., certain individuals following a vegan diet have been observed to experience a low vitamin D intake and exhibit lower serum 25-hydroxyvitamin D levels, especially during winter or in regions with higher latitudes (118). Furthermore, a study by Allès et al. indicated that vegans tend to consume less vitamin D relative to recommended dietary guidelines (3). The recommended daily intake of vitamin D varies, ranging from 600 IU/day to 800 IU/day (124) or approximately 5 μg to 15 μg (52). In particular, Menzel et al. underscore the critical nature of this nutrient, as its deficiency could lead to decreased bone mineral density, increased bone turnover, and an increased risk of premature bone ageing, thus increasing the susceptibility to fractures. This concern is particularly relevant for vegans, who, due to their exclusion of animal-derived foods, face an increased risk of inadequate vitamin D supply, which could lead to adverse effects on bone health (94). Sources of vitamin D include fortified breakfast cereals and non-dairy milk substitutes such as oat, almond and rice beverages. When exposure to the sun and fortified food intake are insufficient to meet dietary requirements, vitamin D supplementation is recommended for individuals of all ages (5).

### 6.4. Omega 3 fatty acids

Omega-3 fatty acids, with a particular focus on alpha-linolenic acid (ALA), play an important role in preventing atherosclerosis and improving lipid profiles through the reduction of inflammation and the mitigation of oxidative stress. ALA, which is an essential fatty acid, acts as a precursor for the synthesis of EPA and DHA (125), but only a small portion is converted to longer-chain fatty acids. Current research indicates that n-3 conversion from short chain to long chain in humans is very limited; ALA converts to EPA at a rate of 5–15%, and < 1% of ALA reliably converts to DHA. Individuals who follow a vegan diet and include no marine foods in their diet will consume ALA because of its wide distribution in plant-sourced foods (126). However, literature suggests there is individual variation in conversion rate of fatty acids, influenced by genetics and dietary habits, including the presence of other fatty acids in the diet. Vegan may be more efficient at n-3 conversion, but this has not been confirmed (127).

According to Menzel et al., the main sources of EPA and DHA, include oily fish, dairy and meat, therefore intake of EPA and DHA among vegans is lower compared to omnivores, due to the omission of these foods from their diet. Their study revealed reduced plasma levels of n-3 fatty acids in vegans (118). ALA sources, such as vegetable oils, cereals, nuts such as walnuts and chia seeds, as well as plant-derived oils such as rapeseed, linseed, canola, and hemp should be included in the well-balanced vegan diet (125, 128). Optimizing the intake of omega-3 fatty acids is crucial for those following a vegan diet (7) as the Adequate Intake (AI) guidelines for n-3 fatty acids suggest a daily intake of 1.1 grams of alpha-linolenic acid for women and 1.6 grams for men (128). An abundance of foods fortified with EPA and/or DHA from either marine or algal sources are now available; called functional foods, examples include soy milks and juices, cooking oils, spreads, snack foods (126).

### 6.5. Calcium

Vegans generally demonstrate lower calcium intake compared to individuals who follow alternative diet patterns such as lacto-ovo-vegetarian diets (52, 118). However, though various plant-based sources offer substantial calcium content, its absorption is negatively influenced by compounds such as oxalates, phytates, and fiber present in vegetables (5). To improve calcium intake, there are a number of interventions that can be implemented. These include promoting the consumption of foods naturally high in calcium, using food processing techniques that could improve calcium content or bioavailability, staple food fortification, and biofortification to produce higher calcium-containing crops (129). Noteworthy calcium-rich plant foods include green leafy vegetables, tofu, tahini, as well as fortified options such as cereals, soy, rice, and nut and fruit beverages. Optimal absorption is observed in low-oxalate vegetables, such as broccoli and kale (5). The WHO recommended dietary allowance for adults ranges from 1,000 to 1,200 mg (130), and meeting this requirement is feasible for vegans by consuming a variety of plant-based foods rich in calcium.

In a comparative study involving various dietary groups, including meat eaters, fish eaters, vegetarians, and vegans, a noticeable increase in fracture rates was observed among vegan participants. This trend appeared to be associated with a significantly lower average calcium intake within the vegan group (128). The study by Menzel et al. offers information on the impact of transitioning from an omnivorous diet to a vegan diet, revealing a reduction in calcium excretion indicative of dietary changes. The study effectively employed 24-h urine samples to accurately assess mineral statuses, unveiling a decrease in calcium excretion among vegans compared to omnivores. This variation in excretion is probably attributed to differences in dietary calcium intake, which is reflected in urinary calcium concentrations (94).

### 6.6. Zinc

Among vegans, decreased plasma zinc levels can contribute to iron deficiency anemia (5). Poor zinc status is most commonly linked to innate immunity and reduced resistance to infections. Dimitra et al. conducted a systematic review that revealed that vegans have the lowest zinc intake compared to groups following various diet habits (52). Furthermore, a study carried out by Allès et al. showcased notable insufficiency of zinc among vegans (3). Zinc serves as a facilitator in iron metabolism and is less readily absorbed from plant-derived sources compared to animal products, which typically contribute about half of the zinc intake. Plant-based sources rich in zinc include wholemeal bread, peas, corn, nuts, carrots, whole grains, wheat germs, soybeans, cabbage, radish, watercress, and legumes (5, 118). Vegans are advised to consume these foods in sufficient amounts to prevent zinc deficiency. Supplementation and the inclusion of fortified breakfast cereals and foods could be crucial for meeting the nutritional needs of individuals following a vegan diet (124). The WHO has established a classification for zinc bioavailability based on the phytic acid: zinc ratio. Ratios below 5 are designated as indicating “high” zinc availability, resulting in 50% absorption (High Bioavailability: Females: 3.0 mg, Males: 4.2 mg). Ratios spanning from 5 to 15 signify “moderate” bioavailability, yielding 30% absorption (Moderate Bioavailability: Females: 4.9 mg, Males: 7.0 mg). Ratios surpassing 15 indicate “low” zinc availability, leading to 15% absorption (Low Bioavailability: Females: 9.8 mg, Males: 14.0 mg). These categories align with recommended zinc intake levels for different gender groups, providing tailored guidance for optimal nutritional adequacy (52, 131). Vegan dietary patterns are categorized as possessing a moderate degree of zinc availability, given that their predominant reliance is not on unrefined, unfermented, or ungerminated cereal grains, or high-extraction-rate flours (131).

### 6.7. Iron

Anemia resulting from iron deficiency is more prevalent among vegans than among omnivores (124). Despite vegans having the potential to achieve a daily iron intake similar to non-vegans, their blood iron and ferritin levels tend to be lower, partly due to the less effective absorption of non-haem iron found in plant-derived foods compared to haem iron from animal sources. This is supported by another study that found higher iron intake among vegans compared to other diets, especially in German vegan women, although the absorption levels did not correspond proportionally to the increased intake (5, 48). Iron sources include legumes, beans, whole grains, whole cereals, dark-green leafy vegetables, fruits, seeds, and nuts (5). Enhanced absorption of non-haem iron is facilitated by ascorbic acid, minor alcohol intake, retinol, and carotenes (5, 132). However, factors such as phytates, tannins/polyphenols, and soy protein inhibit absorption (133). Marrone et al. emphasized that menopausal women among vegans are particularly prone to iron deficiencies. The recommended dietary allowance of iron is established at 8 mg per day for men and 18 mg per day for women (130). There is an iron RDA specific for vegetarians/vegans in the U.S. due to lack of dietary haem iron: 32 milligrams per day for women and 14 milligrams per day for men (119). To combat iron deficiency, fortified foods such as salt, wheat flour, and rice can be incorporated into the diet (14).

Haem iron, which is mainly found in products of animal origin, is not available in a vegan diet. Therefore, people on a vegan diet are not at risk of an excess of this form of iron. Haem iron is a type of iron that is found in haem-containing proteins, such as haemoglobin in the red blood cells and myoglobin in the muscles. It is important to the body because it is necessary for the transport of oxygen from the lungs to tissues and for the storage and transport of oxygen in muscles (134). However, certain types of damage can occur if there is an excess of haem iron or if it is processed incorrectly. One of the main concerns regarding the harmful effects of haem iron is its role in oxidative stress. This is due to its ability to catalyse the formation of reactive oxygen species. Reactive molecules can cause damage to cells, proteins, lipids and nucleic acid (DNA), which can contribute to inflammatory processes (135).

Excess haem iron has been positively associated with non-communicable diseases, including colorectal cancer, type 2 diabetes and cardiovascular mortality (136, 137). In a meta-analysis of prospective cohort studies by Hunnicutt et al. haem iron intake was positively associated with the incidence of coronary heart disease (138). In a prospective cohort study among 539 older Australian men aged 75 years and older, it was shown that higher haem iron intake was independently associated with an increased risk of adverse cardiovascular events, mortality from any cause, congestive heart failure and coronary revascularisation over 5 years (139). The maintenance of the right balance of iron in the body is essential for the maintenance of good health. It is therefore important that not only those who follow a plant-based diet, but also those who eat meat, monitor their iron levels on a regular basis and adjust their diet to include adequate amounts of iron (136).

## 7. Guidelines for adopting a vegan diet

Most guidelines on vegetarian and vegan diets have provided neutral advice on supplementing certain nutrients with plant sources. Guidelines such as those from the United Kingdom, Australia, Belgium, Lebanon, Malaysia and Malta indicate that all nutrients can be obtained from a vegetarian diet, including a vegan diet, by combining a variety of foods and consuming an appropriate amount of calories (140).

For adults aged 18 to 60 years, it is recommended to maintain a total energy intake ranging from 23 to 27 kcal/kg, while those over 60 years should target a range of 19 to 22 kcal/kg. To ensure a balanced carbohydrate and fiber intake, individuals should consume a minimum of 400 g (equivalent to five portions) of fruits and vegetables daily, excluding starchy root and vegetables. Dietary fat intake should be limited to less than 30% of total energy intake, with saturated fats kept below 10% and trans-fats below 1%. It is recommended to substitute saturated and trans fats with unsaturated fats. Protein intake should contribute to approximately 15% of total energy intake. Additionally, it is advised to limit free sugar intake to around 5% of energy and restrict salt intake to 1,500 mg/day when adhering to a vegan diet. To ensure adequate intake of vitamins B12 and D throughout the year can be achieved through vitamin-fortified meals or supplements, and EPA/DHA supplementation (alternate source of EPA/DHA algal oil) is also recommended (4, 60).

People following a strict vegan diet can meet nutrient requirements as long as energy needs are met and an appropriate variety of plant foods are eaten throughout the day (140), and include fortified foods and/or supplements to get adequate amounts of vitamin D and vitamin B12 (4, 60).

## 8. Obstacles to adopting a vegan diet and lifestyle

Individuals adopting a vegan diet can encounter a variety of difficulties. Many people fail to maintain a vegan diet in the long term and give up (133). This can be due to both physical and social obstacles that can affect the maintenance of this eating style.

The first barrier may be insufficient knowledge of the nutrients in a vegan diet, the principles of correct meal composition, or the implementation of supplementation (141). Another potential obstacle may be that veganism requires more dedicated time and commitment to cooking and preparing meals compared to meat-based options. This can be complicated by the perception that such a diet is tasteless and can easily become monotonous (142). Currently, the market offers a variety of meat and dairy substitutes that do not require much time to prepare (143). However, most plant-based meat alternatives are classified as ultra-processed foods (UPF) (144). Higher UPF intake is associated with an increased risk of obesity and diet-related non-communicable diseases (such as cardiovascular disease, diabetes, cancer) and even higher mortality (145).In addition, some people become attached to the taste of meat products, which can make it difficult to change eating habits, especially at the initial stage of changing their diet (146). One potential obstacle could be the difficulty of access to high-quality, fresh plant products and their higher cost compared to animal products (147). According to Fehér et al., meat prices have a clear impact on the willingness to switch to a plant-based diet (141).

Social pressure, especially from family, loved ones, and friends, is generally considered a significant influence on meat consumption. Some individuals may fear switching to a vegan diet because they expect stigma and ostracism from significant others (148). Adopting a vegan diet can affect family relationships, which may explain why those who choose this diet often experience a lack of understanding or even negative reactions from family members who consume animal products (149). Situations described as one of many factors seem to have an impact on mental health of people following a vegan diet, but scientific views on the impact of plant-based nutrition on mental health are divided. In the Dobersek et al. systematic review, the majority of studies, especially the higher-quality studies, found that people who avoided eating meat had significantly higher rates or risks of depression, anxiety and/or self-injurious behaviour (150). Forestell and Nezlek indicated that people who follow a plant-based diet are more likely to be depressed (151). Furthermore, women on a vegan diet are more likely than men to have disordered eating attitudes and practices (152).

One potential danger associated with a vegan diet is the risk of malnutrition, which can occur in individuals if the diet is not balanced and does not provide the body with sufficient essential nutrients. Although our review does not include studies on pregnant and breastfeeding women, it is worth mentioning that this is very important in the context of the impact on the fetus and child. According to international guidelines, a plant-based diet during pregnancy and lactation requires a high level of awareness to ensure complete intakes of essential key nutrients and vitamin supplements. Maternal undernutrition can potentially alter fetal growth trajectories by altering placental weight and nutrient transfer capacity, depending on the severity and timing of nutrient deprivation. Maternal malnutrition leading to vitamin B12, vitamin D, calcium and DHA deficiencies during lactation may contribute to low levels of these nutrients in breast milk (153).

Despite the barriers mentioned, many people successfully start and follow a vegan diet long-term and reap the health, ethical, and environmental benefits. Given the current growing interest in plant-based diets among the general population, it is crucial to understand both the barriers, risks, and benefits of such diets among clinicians, policy makers, and the general population (154). A food policy that combines health, sustainability, and affordability can effectively accelerate the promotion of plant-based diets and support the achievement of mitigation targets for potential barriers.

## 9. Conclusion

It is believed that a well-planned vegan diet, when combined with a healthy and active lifestyle, is a viable choice for healthy adults, especially those who follow it. This is because chronic diseases are significantly more common than they used to be and various strategies to address these public health challenges are insufficient.

Numerous studies have demonstrated the validity of this claim and any doubts have been attributed to an inadequately designed vegan diet, which is a potential problem with any kind of diet (such as omnivorous). As people age, their caloric needs tend to decrease, while their requirements for specific nutrients may increase.

A well-planned vegan diet must include adequate calories and nutrients, as well as the necessary supplements, such as vitamin B12 and vitamin D. To reduce the risk of vitamin deficiencies, fortified foods should be consumed by adults and the general population. Vegans are strongly encouraged to consult their doctors or dietitians before switching to a vegan diet.

Furthermore, the implementation of well-designed vegan diets and lifestyles requires greater awareness, greater social responsibility, and government involvement to ensure the fair cost of vegan food products. It should be emphasized that the advantages and drawbacks of vegan diets for adults are not fully covered in this review. The precise processes through which vegan diets work in many chronic diseases require further studies. Lastly, future studies should use large sample sizes that are accurately representative of the adult population.

## Author contributions

EŁ: Conceptualization, Methodology, Resources, Writing – original draft, Writing – review & editing. FB: Conceptualization, Data curation, Methodology, Writing – original draft, Writing – review & editing. MZ: Data curation, Writing – original draft, Writing – review & editing. KD: Data curation, Resources, Writing – original draft, Writing – review & editing. AB: Data curation, Writing – review & editing. ŁO: Data curation, Writing – review & editing. AS: Data curation, Resources, Writing – review & editing.
