# Corrigendum: Vegan diet: nutritional components, implementation, and effects on adults' health

**DOI:** 10.3389/fnut.2023.1354336

**Published:** 2024-01-05

**Authors:** Edyta Łuszczki, Faustina Boakye, Magdalena Zielińska, Katarzyna Dereń, Anna Bartosiewicz, Łukasz Oleksy, Artur Stolarczyk

**Affiliations:** ^1^Institute of Health Sciences, Medical College of Rzeszów University, Rzeszów, Poland; ^2^Faculty of Health Sciences, Department of Physiotherapy, Jagiellonian University Medical College, Kraków, Poland; ^3^Orthopedic and Rehabilitation Department, Medical University of Warsaw, Warsaw, Poland

**Keywords:** bioactive compounds, health, plant-based sources, prevention, vegan diet

In the published article, there was an error in affiliation 2. Instead of “Centre des Sciences du Goût et de l'Alimentation, AgroSup Dijon, CNRS, INRA, Université Bourgogne – Franche-Comté, Dijon, France”, it should be “Institute of Health Sciences, Medical College of Rzeszów University, Rzeszów, Poland.”

The authors apologize for this error and state that this does not change the scientific conclusions of the article in any way. The original article has been updated.

